# On the Sensitivity of Granger Causality to Errors‐In‐Variables, Linear Transformations and Subsampling

**DOI:** 10.1111/jtsa.12430

**Published:** 2018-09-23

**Authors:** Brian D.O. Anderson, Manfred Deistler, Jean‐Marie Dufour

**Affiliations:** ^1^ School of Automation Hangzhou Dianzi University Hangzhou China; ^2^ Research School of Engineering, ANU College of Engineering and Computer Science Australian National University Acton Australia; ^3^ Data61‐CSIRO Canberra Australia; ^4^ Technische Universität Wien, Institut für Stochastik und Wirtschaftsmathematik, Forschungsgruppe Ökonometrie und Systemtheorie Wien Austria; ^5^ Department of Economics McGill University Montréal Canada; ^6^ Centre interuniversitaire de recherche en analyse des organisations (CIRANO) Montréal Canada; ^7^ Centre interuniversitaire de recherche en ‘economie quantitative (CIREQ) Montréal Canada; ^8^ Institute for Advanced Studies Vienna Austria

**Keywords:** Granger causality, sensitivity, signal‐to‐noise ratio, errors‐in‐variables, measurement errors, filtering, subsampling

## Abstract

This article studies the sensitivity of Granger causality to the addition of noise, the introduction of subsampling, and the application of causal invertible filters to weakly stationary processes. Using canonical spectral factors and Wold decompositions, we give general conditions under which additive noise or filtering distorts Granger‐causal properties by inducing (spurious) Granger causality, as well as conditions under which it does not. For the errors‐in‐variables case, we give a continuity result, which implies that: a ‘small’ noise‐to‐signal ratio entails ‘small’ distortions in Granger causality. On filtering, we give general necessary and sufficient conditions under which ‘spurious’ causal relations between (vector) time series are not induced by linear transformations of the variables involved. This also yields transformations (or filters) which can eliminate Granger causality from one vector to another one. In a number of cases, we clarify results in the existing literature, with a number of calculations streamlining some existing approaches.

## 1 Introduction

Granger causality is one of the most important concepts for the analysis of the structure of multivariate time series. Accordingly, the original article of Granger (1969) triggered a substantial number of publications, see for example Sims (1972), Pierce and Haugh (1977), Granger, (1980, 1988) , Geweke, (1982, 1984, 1984) , Boudjellaba et al. (1992), Dufour and Tessier (1993), Dufour and Renault (1998), Al‐Sadoon (2014) and the references therein. Here we deal with an aspect of Granger causality, namely the sensitivity of Granger causality relations with respect to measurement errors (or errors‐in‐variables) in the observations. In particular, we study the effect of additive noise on Granger causality in the context of a general weakly stationary multivariate model, especially in view of finding when spurious causality could appear, and when properties of non‐causality are unaffected by measurement errors.

The problem of measurement errors is a classical issue in statistical theory; see for example the reviews of Fuller (1987), Wansbeek and Meijer (2000), Carroll et al. (2006), Gustafson (2003), and Buonaccorsi (2010). However, except for the early article by Newbold (1978), there is surprisingly little work on the effect of errors‐in‐variables on Granger causality. In this work, Newbold showed that measurement errors can produce artificial feedback in the noisy series, even though no such feedback is present before noise is superimposed. No general characterization of cases where such spurious causality could appear was however provided. From a wider perspective, several authors have emphasized that the addition of noise to time series (errors‐in‐variables) can substantially modify the structure of the series, leading to distortions and identification problems; see for example the literature reviewed by Maravall (1979), Anderson and Deistler (1984), Anderson (1985), Deistler and Anderson (1989), and Scherrer and Deistler (1998). Note also that measurement errors may give rise to additive ‘outliers’ which may strongly influence the results of estimation and testing procedures.

The question of the sensitivity to measurement errors is quite distinct for the effect of aggregation and subsampling, for these transformations typically considerably reduce the effective sample size. For work on the latter problems, the reader may consult Tiao and Wei (1976), Wallis (1974), Sims (1974), Wei (1982), Hylleberg (1986), Marcellino (1999), Kaiser and Maravall (2001), Breitung and Swanson (2002), McCrorie and Chambers (2006) Barnett and Seth, 2011 (2011, 2015, 2017) , Smirnov and Bezruchko (2012), Gong et al. (2015), Ghysels et al. (2016), and the references in the survey of Silvestrini and Veredas (2008).

Errors‐in‐variables can be interpreted as missing variables: if the noise were observable, it could be included as an additional variable, and different conclusions can emerge. As previously observed by several authors (see Hsiao, 1982; Lütktepohl, 1982; Dufour and Renault, 1998; Triacca, 1998, 2000), causality properties in the sense of Wiener–Granger depend crucially on the information set considered, which can affect both the sheer presence of causality (or non‐causality) and causality measures (Geweke, 1982; Dufour and Taamouti, 2010; Dufour et al. 2012). Of course, the central difficulty remains that noise is typically unobserved. In this article, we revisit the questions of the effect of (unobserved) additive noise on Granger (non‐)causality, and using the same tools, rapidly traverse also issues of the effects of filtering and subsampling.

Let X=(X(t)|t∈Z), $X(t):\Omega \to R^{d}$, be a vector process of dimension *d* with finite second moments, where Z represents the integers and R the real numbers. We assume that *X* is weakly stationary, centered (i.e., E[X(t)]=0) and Gaussian, with a full‐rank rational spectral density.^1^ We postulate that the process *X* can be regarded as a juxtaposition of two subprocesses $X=(X_{A}^{⊤}\;$ $X_{B}^{⊤}){}^{⊤}$. The broad question we study is whether the past values of *X*_*A*_ improve the prediction of *X*_*B*_. To be more precise, one says that *X*_*A*_ does not Granger cause *X*_*B*_ if

(1) $$E[X_{B}(t)\;|\;X_{A}(s),X_{B}(s):s<t]=E[X_{B}(t)\;|\;X_{B}(s):s<t]$$

or equivalently

(2) $$\mathrm{Var}[X_{B}(t)\;|\;X_{A}(s),X_{B}(s):s<t]=\mathrm{Var}[X_{B}(t)\;|\;X_{B}(s):s<t].$$

Here $E[X_{B}(t)\;|\;X_{A}(s),$ *X*_*B*_(*s*):*s* < *t*] denotes the conditional expectation of *X*_*B*_(*t*)[given the variables *X*_*A*_(*s*),*X*_*B*_(*s*) such that *s* < *t* (and similarly elsewhere)], and Var the variance of the one‐step‐ahead forecast error. If inequality holds in (1) and (2), one says that *X*_*A*_ (Granger) causes *X*_*B*_. Granger (1969) in addition introduced the notion of ‘instantaneous causality’ , meaning that the approximation of *X*_*B*_(*t*) can be more accurately achieved if *X*_*A*_(*t*) is known:

(3) $$E[X_{B}(t)\;|\;X_{A}(t),X_{A}(s),X_{B}(s):s<t]\neq E[X_{B}(t)\;|\;X_{A}(s),X_{B}(s):s<t]\;;$$

for further discussion of this notion, see Pierce and Haugh (1977) and Granger (1988). The assumption of second‐order stationarity is clearly restrictive, but is standard in the Granger‐causality literature. Further, general characterizations of non‐causality are typically little affected when common forms of forms of non‐stationarity – such deterministic time trends and integration) – are allowed; see, for example, Dufour and Renault (1998) and Dufour et al. (2006).

It is clear from the above definitions that Granger causality depends on the vector *X* considered and on the way *X* is split into subvectors *X*_*A*_ and *X*_*B*_. Such choices (which are of course finite in number) depend on the context: which variables are of interest, and the objectives of the analysis. For example, *X*_*A*_ can represent policy instruments (e.g., fiscal and monetary variables) or leading indicators of economic activity, and *X*_*B*_ economic outcomes (e.g., national income, unemployment, etc.): the nature of the variables often provides a natural criterion for splitting *X* into subvectors. Clearly, the causal structure of a time series should in general depend on such choices. However, the question remains whether apparently less fundamental features, such as contamination by noise and various linear transformations, including filtering and subsampling, can affect the causal properties of a time series.

This article studies the sensitivity of Granger causality to the addition of noise, the application of causal invertible filters, and subsampling in weakly stationary processes. We give general conditions under which additive noise or filtering creates distortions by inducing (spurious) Granger causality, as well as conditions under which it does not. Even though additive noise and filtering can in general produce spurious Granger causality, there is a remarkably wide range of cases where it does not. For example, if the ‘caused variable’ *X*_*B*_ is not noisy, noise added to the ‘causal variable’ *X*_*A*_ cannot induce spurious Granger causality from *X*_*A*_ to *X*_*B*_. This covers cases where lagged values of *X*_*A*_ are contaminated by noise, and *X*_*B*_ does Granger‐cause *X*_*A*_. We also give a continuity result which entails a ‘small’ noise‐to‐signal ratio in measurement errors entails ‘small’ distortions in Granger causality. In a number of cases, we clarify results in the existing literature, with a number of calculations streamlining some existing approaches.

We also consider the effects of linear transformations, filtering and subsampling. In particular, we give general necessary and sufficient conditions under which ‘spurious’ causal relations between (vector) time series are not be induced by linear transformations of the variables involved. This also yields linear transformations (or filters) which can eliminate Granger causality from one vector to another one.

Section 2 summarizes a collection of known results available for the characterization of Granger causality, using canonical spectral factors, Wold decompositions and spectra. In Section 3, we establish some connections not clearly stated in the earlier literature, which are useful for studying causality in the presence of measurement errors. These include: a general lower bound on the conditional variance of the sum of two processes, and some general relations between Granger causality and instantaneous causality. In Section 4, we study the effect of measurement errors on Granger non‐causality. Section 5 provides the continuity result in terms of signal‐to‐noise ratio. The effects of linear transformations, filtering and subsampling are studied in Sections 6 and 7. Section 8 offers some concluding remarks. Proofs appear in the Appendix.

## 2 Characterizations of Granger Causality

We review some classical characterizations of Granger causality which will be useful for studying the effect of errors‐in‐variables. We first record some notational conventions associated with rational (matrix) transfer functions (see e.g. Rozanov, 1967; Hannan and Deistler, (Hannan and Deistler, 2012)). We emphasize the use of spectral methods, for which Geweke, (1982, 1984, 1984) was an early promoter in the context of analyzing Granger–Wiener causality.

A rational transfer function is called *stable* if its poles are outside the unit circle, and it is called *miniphase* or *minimum phase* if its zeros are outside the unit circle. If we commence from a rational spectral density Φ_*XX*_(*z*), z∈C, which is positive definite everywhere on the unit circle, there is a spectral factorization

(4) $$\Phi_{XX}(z)=W(z)\;Q\;W^{⊤}(z^{-1})$$

in which the spectral factor *W*(*z*) is a square real rational, stable and miniphase, transfer function and *Q* is positive definite symmetric; see Rozanov (1967), Hannan and Deistler (2012). *W*(*z*) defines a linear filter on replacing *z* by the backshift operator *L* (i.e., *LX*(*t*): = *X*(*t* − 1)). The notation *W*(*z*) allows one to study the properties of lag operators in terms of the analytical properties of functions of a complex variable z∈C. Under the normalization *W*(0) = *I*_*d*_, *W*(*z*) and *Q* are unique. We also consider the following assumption.

Assumption 1 **(Full rank stationary process with no spectral zero on the unit circle)** $X=(X_{A}^{⊤}\;$ $X_{B}^{⊤}){}^{⊤}$ is a real full‐rank stationary stochastic process in $R^{d}$, with rational spectrum Φ_*XX*_(*z*) having no zero on the unit circle, such that (4) is satisfied, *W*(0) = *I*_*d*_, and

(5) $$W(z)=\left[\begin{array}{cc} W_{11}(z) & W_{12}(z)\\ W_{21}(z) & W_{22}(z) \end{array}\right]\;\;Q=\left[\begin{array}{cc} Q_{11} & Q_{12}\\ Q_{21} & Q_{22} \end{array}\right]$$

are partitioned conformably with $X=(X_{A}^{⊤}\;$ $X_{B}^{⊤}){}^{⊤}$.

The above assumption entails that *X*(*t*) has both a moving average (Wold) representation

(6) X(t)=W(L)ϵ(t)

and an autoregressive representation

(7) Π(L)X(t)=ϵ(t)

where det[W(z)]≠0 and det[Π(z)]≠0 for $\left|z\right|<1,$ Π(*z*) = *W*(*z*)^−1^, and *ϵ*(*t*) = [*ϵ*_*A*_(*t*)^⊤^ *ϵ*_*B*_(*t*)^⊤^]^⊤^ represents the innovations of the process, partitioned conformably with $X=(X_{A}^{⊤}\;$ $X_{B}^{⊤}){}^{⊤}$. The following theorems provide characterizations of Granger causality; see Sims (1972), Pierce and Haugh (1977), Geweke, (1982, 1984, 1984) , Boudjellaba et al. (1992), Dufour and Tessier (1993), Dufour and Renault (1998). The first one is based on the structure of the spectral factor matrix *W*(*z*).

Theorem 1 **(Canonical spectral factor characterization of Granger causality)** Suppose Assumption 1 holds. Then the following two conditions are equivalent:

- *X*_*A*_ does not Granger cause *X*_*B*_;
- *W*_21_(*z*) = 0.

The following conditions are also equivalent:

- *X*_*A*_ neither Granger causes *X*_*B*_, nor does it cause *X*_*B*_ instantaneously;
- *W*_21_(*z*) = 0 and *Q* is block diagonal (i.e. $Q_{12}=Q_{21}^{⊤}=0)$.

The intuition behind the above claim is the following. Let the innovation process be denoted by *ϵ*(*t*) = [*ϵ*_*A*_(*t*)^⊤^ *ϵ*_*B*_(*t*)^⊤^]^⊤^ with *ϵ*_*A*_ and *ϵ*_*B*_ two independent white noise processes. When *W*_21_(*z*) = 0, we have:

(8) $$\begin{array}{lll} X_{A}(t) & = & W_{11}(L)\epsilon_{A}(t)+W_{12}(L)\epsilon_{B}(t)\;\\ X_{B}(t) & = & W_{22}(L)\epsilon_{B}(t). \end{array}$$

It is intuitively reasonable to conclude from these equations that knowledge of the *X*_*A*_ process up till time *t* − 1 will not be of help in determining the *ϵ*_*B*_ process and thus the *X*_*B*_ process. Spectral approaches for Granger causality analysis were emphasized in the seminal work of Geweke, (1982, 1984, 1984) .

For completeness, we note a further characterization of Granger causality, which follows from the above.

Theorem 2 **(AR characterization of Granger causality)** Suppose Assumption 1 holds, and *X*(*t*) has the (possibly infinite) autoregressive representation

(9) $$X(t)=\sum_{i=1}^{\infty }A_{i}X(t-i)+\epsilon (t)\;\;A_{i}=\left[\begin{array}{cc} A_{i11} & A_{i12}\\ A_{i21} & A_{i22} \end{array}\right]\;\;\mathrm{Var}[\epsilon (t)]=\left[\begin{array}{cc} \Sigma_{11} & \Sigma_{12}\\ \Sigma_{21} & \Sigma_{22} \end{array}\right]$$

where the *A*_*i*_ and the covariance matrix Var[*ϵ*(*t*)] of the innovations sequence *ϵ*(*t*) are partitioned conformably with $X=(X_{A}^{⊤}\;$ $X_{B}^{⊤}){}^{⊤}$. Then *X*_*A*_ does not Granger cause *X*_*B*_ if and only if *A*_*i*21_ = 0 for all *i* ≥ 1. In addition, *X*_*A*_ neither Granger causes *X*_*B*_, nor does it cause *X*_*B*_ instantaneously if and only if *A*_*i*21_ = 0 for all *i* ≥ 1 and $\Sigma_{12}=\Sigma_{21}^{⊤}=0$.

Theorems 1 and 2 give characterizations of the absence of causality based on the spectral factor and infinite AR representations (the latter is obtained from the inverse of the spectral factor). Sims (1972) gave an additional characterization (for *d* = 2), based on Wiener filtering ideas, where no factorization is required. Let the spectral density Φ_*XX*_ be partitioned conformably with $X=(X_{A}^{⊤}\;$ $X_{B}^{⊤}){}^{⊤}$ as

(10) $$\Phi_{XX}=\left[\begin{array}{cc} \Phi_{AA} & \Phi_{AB}\\ \Phi_{BA} & \Phi_{BB} \end{array}\right].$$

Then we have the following spectral characterization of non‐causality.

Theorem 3 **(Transfer function characterization of Granger causality)** Suppose Assumption 1 holds, and let Φ_*XX*_ be partitioned as in (). Then, the following conditions are equivalent:

- *X*_*A*_ does not Granger cause *X*_*B*_;
- $\Phi_{AB}(z)\Phi_{BB}^{-1}(z)$ is a stable transfer function.

The following conditions are also equivalent:

- *X*_*A*_ neither Granger causes *X*_*B*_ nor does it cause *X*_*B*_ instantaneously;
- $\Phi_{AB}(z)\Phi_{BB}^{-1}(z)$ is a stable transfer function assuming the value 0 at *z* = 0.

Remark 1 The above theorem can be viewed as an extension of the corresponding theorem given by (Sims, 1972, Theorem 2) in the special case where *d* = 2. Theorem 3 allows for *d* ≥ 2, and covers instantaneous causality as well.^2^ We are not contending that the characterization of this theorem is necessarily attractive from a computational point of view. As later parts of the article show though, the result is of theoretical interest, in that it can be applied to give rapid derivations of the sensitivity properties associated with Granger causality.

Remark 2 The transfer function $\Phi_{AB}(z)\Phi_{BB}^{-1}(z)$ is the transfer function of the optimum two‐sided Wiener filter for approximating the process *X*_*A*_ from the process *X*_*B*_; the two‐sided aspect refers both to the fact that the transfer function has a Laurent series expansion with both negative and positive powers of *z*, and to the related fact that *X*_*A*_(*t*) is being approximated from *X*_*B*_(*s*),−*∞* < *s* < *∞*, that is, from the past and future of *X*_*B*_. If the two‐sided transfer function in a particular case is causally one‐sided, then future values of *X*_*B*_ are irrelevant in approximating current values of *X*_*A*_. This will be the case if past values of *X*_*A*_ do not affect present or future values of *X*_*B*_.

Remark 3 It is important to note that the characterizations given in this section hold for series in discrete time observed at a given frequency. They are directly applicable to continuous time series, and modifications arise typically when the series are transformed or filtered. The effect of such transformations will be considered in sections 6 and 7 below.

## 3 Directions of Granger Causality

In the literature, one finds remarkable similarity between conditions said to capture ‘*X*_*A*_ does not cause *X*_*B*_’ and ‘*X*_*B*_ causes *X*_*A*_’ and similar pairings. To study the effect of errors‐in‐variables on causality, we establish in this section some connections not clearly stated in the earlier literature. We start with the following preliminary result.

Lemma 1 Let *X* and *Y* be two independent stationary stochastic processes with spectral densities. Let *Z* = *X* + *Y*. Then the covariance matrix of the one step prediction error in approximating *Z*(*t* + 1) from *Z*(*s*),*s* ≤ *t* is bounded from below by the sum of the covariance matrices of the one step prediction error in approximating *X*(*t* + 1) from *X*(*s*),*s* ≤ *t* and in approximating *Y*(*t* + 1) from *Y*(*s*),*s* ≤ *t*:

(11) Var[Z(t)|Z(s):s<t]≥Var[X(t)|X(s):s<t]+Var[Y(t)|Y(s):s<t].

Now we spell out the following relations between Granger causality and instantaneous causality.

Theorem 4 Adopt the same hypothesis as in Theorem 1. Suppose *X*_*A*_ does not Granger cause *X*_*B*_ nor does it cause *X*_*B*_ instantaneously. Then either the two processes are independent, or *X*_*B*_ Granger causes *X*_*A*_. Further, suppose alternatively that *X*_*A*_ does not cause *X*_*B*_. Then, either the two processes are independent, or *X*_*B*_ Granger causes *X*_*A*_, or *X*_*B*_ causes *X*_*A*_ instantaneously.

Note that neither claim of the theorem goes in the reverse direction. This is because it is possible that both *X*_*A*_ Granger causes *X*_*B*_ and simultaneously *X*_*B*_ Granger causes *X*_*A*_. Such a situation will generally arise when the canonical spectral factor *W* is not triangular (or diagonal), as in the following example:

(12) $$X_{A}(t)=\epsilon_{A}(t)+X_{B}(t-1)\;\;X_{B}(t)=\frac{1}{2}X_{A}(t-1)+\epsilon_{B}(t).$$

Here, *ϵ*_*A*_,*ϵ*_*B*_ are independent white noise processes with variances *Q*_*A*_,*Q*_*B*_. One can verify that

(13) $$\left[\begin{array}{c} X_{A}(t)\\ X_{B}(t) \end{array}\right]=\frac{1}{1+(1/2)L^{2}}\left[\begin{array}{cc} 1 & L\\ (1/2)L & 1 \end{array}\right]\left[\begin{array}{c} \epsilon_{A}(t)\\ \epsilon_{B}(t) \end{array}\right]$$

and the transfer function matrix is easily verified to be stable and minimum phase, assuming the value *I* when *z* = 0. It is easily checked that Var[*X*_*A*_(*t*)|*X*_*A*_(*s*),*X*_*B*_(*s*),*s* < *t*] = *Q*_*A*_,Var[*X*_*B*_(*t*)|*X*_*B*_(*s*),*X*_*A*_(*s*),*s* < *t*] = *Q*_*B*_ while Var[*X*_*A*_(*t*)|*X*_*A*_(*s*),*s* < *t*] > *Q*_*A*_,Var[*X*_*B*_(*t*)|*X*_*B*_(*s*),*s* < *t*] > *Q*_*B*_ by a similar argument to that used in the proof of Theorem 4.

## 4 Additive Noise and Granger Causality

We consider the effect of additive noise on Granger causality (compare with Anderson and Deistler (1984) and Anderson (1985)). Our starting point, again, is the full‐rank stationary process $X={\left[X_{A}^{⊤}\;X_{B}^{⊤}\right]}^{⊤}$ with rational spectral density.

Suppose that *X*_*A*_ does not Granger cause *X*_*B*_. Suppose further that the processes *X*_*A*_,*X*_*B*_ are both contaminated by stationary colored additive noise processes *N*_*A*_,*N*_*B*_ with rational spectral densities, which are independent of each other and of the processes *X*_*A*_,*X*_*B*_. Then one can ask whether it is now true that the process ${\bar{X}}_{A}=X_{A}+N_{A}$ does not Granger cause the process ${\bar{X}}_{B}=X_{B}+N_{B}$. Perhaps of equal if not greater interest is the associated question: suppose that ${\bar{X}}_{A},$ ${\bar{X}}_{B}$ are regarded as noisy measurements of underlying processes *X*_*A*_,*X*_*B*_ and that analysis of measurement data reveals that ${\bar{X}}_{A}$ does not cause ${\bar{X}}_{B}$. Can one conclude then that *X*_*A*_ does not Granger cause *X*_*B*_?

In the next section, we will construct an example showing that the answer to the first question is generally no, a conclusion that is perhaps not counterintuitive since non‐causality corresponds to zero restrictions. In the following section, we show how the Sims (1972) characterization of the absence of Granger causality summarized in Theorem 3 reveals that the claim remains valid if the contaminating noise *N*_*B*_ is zero, and this is generically a necessary condition for the claim to hold. There is no similar requirement on the noise *N*_*A*_. In a article of Solo (2007), several important questions are raised about the sensitivity of Granger causality (or its absence) to changes in the underlying assumptions. We consider one of these, namely the effect of additive noise. Our results differ from those obtained in Solo (2007).^3^ We first study the stationary full‐rank vector process *X* = {*X*(*t*):t∈Z} such that *X*(*t*) = [*X*_*A*_(*t*)^⊤^ *X*_*B*_(*t*)^⊤^]^⊤^ can be regarded as the juxtaposition of two subprocesses *X*_*A*_ and *X*_*B*_. Suppose that *X*_*A*_ does not Granger cause *X*_*B*_ nor does it cause *X*_*B*_ instantaneously.

### 4.1 Noise‐Induced Granger Causality

We will now introduce the promised example. To define the *X*_*A*_,*X*_*B*_ processes where *X*_*A*_ does not Granger cause *X*_*B*_ nor does it cause *X*_*B*_ instantaneously, following Theorem 1 we shall choose an upper triangular canonical spectral factor. The two processes are scalar, and we assume

(14) $$W(z)=\left[\begin{array}{cc} 1+\frac{1}{2}z & z\\ 0 & 1+\frac{1}{2}z \end{array}\right]$$

and we further assume the innovations covariance *Q* is the identity matrix. An easy calculation delivers

(15) $$\Phi_{XX}=\left[\begin{array}{cc} \Phi_{AA} & \Phi_{AB}\\ \Phi_{BA} & \Phi_{BB} \end{array}\right]=\left[\begin{array}{cc} \frac{9}{4}+\frac{1}{2}z+\frac{1}{2}z^{-1} & \frac{1}{2}+z\\ \frac{1}{2}+z^{-1} & \frac{5}{4}+\frac{1}{2}z+\frac{1}{2}z^{-1} \end{array}\right].$$

Now assume that additive noise with a white spectrum of intensity $\frac{3}{4}$ is added to *X*_*B*_, to produce a new process ${\bar{X}}_{B}$, while no noise is added to *X*_*A*_. The cross spectrum between *X*_*A*_ and *X*_*B*_ is unaffected. So the new joint spectral matrix is

(16) $$\Phi_{\bar{X}\bar{X}}=\left[\begin{array}{cc} \Phi_{AA} & \Phi_{A\bar{B}}\\ \Phi_{\bar{B}A} & \Phi_{\bar{B}\bar{B}} \end{array}\right]=\left[\begin{array}{cc} \frac{9}{4}+\frac{1}{2}z+\frac{1}{2}z^{-1} & \frac{1}{2}+z\\ \frac{1}{2}+z^{-1} & 2+\frac{1}{2}z+\frac{1}{2}z^{-1} \end{array}\right].$$

If it were true that ${\bar{X}}_{A}$ does not Granger cause ${\bar{X}}_{B}$, nor cause ${\bar{X}}_{B}$ instantaneously, then this matrix would need to have a canonical spectral factor $\bar{W}(z)$ say, which like *W*(*z*) is upper triangular with $\bar{W}(0)=I$, and an associated innovations covariance matrix which is diagonal. To derive a contradiction, let us assume this to be the case and find $\bar{W}(z)$. The upper triangularity implies that the (2,2) term ${\bar{W}}_{22}$ of $\bar{W}(z)$ must satisfy ${\bar{W}}_{22}(0)=I$ and

(17) $$\Phi_{\bar{B}\bar{B}}(z)={\bar{W}}_{22}(z){\bar{Q}}_{2}{\bar{W}}_{22}(z^{-1})\;$$

which means that ${\bar{W}}_{22}(z)$ itself is a canonical spectral factor, for $\Phi_{\bar{B}\bar{B}}(z)$. One can easily verify that

(18) $$2+\frac{1}{2}z+\frac{1}{2}z^{-1}=\left(1+\frac{\sqrt{3}}{2}\right)\left(1+\frac{z}{2+\sqrt{3}}\right)\left(1+\frac{z^{-1}}{2+\sqrt{3}}\right)\;\;$$

so we see that

(19) $${\bar{W}}_{22}(z)=1+\frac{z}{2+\sqrt{3}},\;\;{\bar{Q}}_{2}=1+\frac{\sqrt{3}}{2}$$

Now consider the (1,2) entry $\Phi_{A\bar{B}}(z)$ of the spectrum. From the fact that when $\bar{W}(z)$ is triangular, we have that

(20) $$\Phi_{A\bar{B}}(z)={\bar{W}}_{12}(z){\bar{Q}}_{2}{\bar{W}}_{22}(z^{-1})$$

from which we obtain

(21) $$\frac{1}{2}+z={\bar{W}}_{12}(z)\left(1+\frac{\sqrt{3}}{2}\right)\left(1+\frac{z^{-1}}{2+\sqrt{3}}\right).$$

It is easy to see that ${\bar{W}}_{12}(z)$ has a pole at $-1/(2+\sqrt{3})$, which is inside the unit circle. This is a contradiction to the requirement on the poles of a canonical spectral factor that they should all lie outside the unit circle.

### 4.2 Spectral Characterization of Noise‐Induced Granger Causality

It is now straightforward to understand the effect of adding noise to the processes *X*_*A*_,*X*_*B*_ on the property that *X*_*A*_ does not Granger cause *X*_*B*_. Suppose as before that *N*_*A*_,*N*_*B*_ are two processes, independent of *X*_*A*_,*X*_*B*_ and each other, and added to *X*_*A*_,*X*_*B*_ to yield new processes ${\bar{X}}_{A}=X_{A}+N_{A},$ ${\bar{X}}_{B}=X_{B}+N_{B}$. The outcome is that

(22) $$\Phi_{Ā\bar{B}}=\Phi_{AB}\;\;\Phi_{\bar{B}\bar{B}}=\Phi_{BB}+\Phi_{N_{B}N_{B}}.$$

The absence of Granger causality will carry over, that is, ${\bar{X}}_{A}$ will not Granger cause ${\bar{X}}_{B}$ if and only if (by Theorem 3), $\Phi_{Ā\bar{B}}\Phi_{\bar{B}\bar{B}}^{-1}$ is a stable transfer function. If there is noise on the process *X*_*A*_ but not the process *X*_*B*_, the result is immediate that absence of causality continues to hold; the same transfer function fraction in fact arises, for $\Phi_{AB}\Phi_{BB}^{-1}=\Phi_{Ā\bar{B}}\Phi_{\bar{B}\bar{B}}^{-1}$ . On the other hand, if there is noise on the process *X*_*B*_, for ‘almost all’ spectra of $\Phi_{N_{B}N_{B}}$, including certainly a white spectrum, unless Φ_*BB*_ is itself white, the zeros of $\Phi_{BB}+\Phi_{N_{B}N_{B}}$ will differ from those of Φ_*BB*_ and not be the same as the poles of $\Phi_{Ā\bar{B}}=\Phi_{AB}$. So *the cancellation of unstable pole‐zero pairs in forming the fraction will no longer occur* and the absence of Granger causality will then be lost.

Now let us postulate that processes ${\bar{X}}_{A},$ ${\bar{X}}_{B}$ are measured and found to have the property that ${\bar{X}}_{A}$ does not Granger cause ${\bar{X}}_{B}$; these processes are assumed to be noisy versions of underlying processes *X*_*A*_,*X*_*B*_, with the additive noise processes being independent of each other and the underlying *X*_*A*_,*X*_*B*_ processes. Ultimate interest lies in saying whether or not *X*_*A*_ Granger causes *X*_*B*_. Then the above argument shows that if we knew that there was no noise perturbing *X*_*B*_, processing of the noisy measurements would allow answering of the question. On the other hand, if there is noise perturbing *X*_*B*_, one could not infer from the presence or absence of a causality property involving ${\bar{X}}_{A},$ ${\bar{X}}_{B}$ the corresponding property for *X*_*A*_,*X*_*B*_. The noise process *N*_*B*_ would need to have a specialized spectrum for absence of causality in the noisy case to imply it in the noiseless case. Note that there is no adjustment to the conclusions which arises in the special case of the noise process *N*_*B*_ being white.

The results above are summed up in Theorem 5.

Theorem 5 Adopt the same hypothesis as in Theorem 1. Let *N*_*A*_,*N*_*B*_ be two stationary processes with rational spectra, with the same dimensions as *X*_*A*_,*X*_*B*_ respectively, where *X*,*N*_*A*_,*N*_*B*_ mutually independent, and set ${\bar{X}}_{A}=X_{A}+N_{A},$ ${\bar{X}}_{B}=X_{B}+N_{B}$.

- If *N*_*B*_ = 0, then
  $$X_{A}\;\text{does not Granger cause}\;X_{B}\;\text{if and only if}\;{\bar{X}}_{A}\;\text{does not Granger cause}\;{\bar{X}}_{B}.$$
- If *N*_*B*_ ≠ 0 and not all the unstable zeros of $\Phi_{BB}+\Phi_{N_{B}N_{B}}$ cancel (unstable) zeros of Φ_*AB*_, we have the following implications:
      (a) if *X*_*A*_ does not Granger cause *X*_*B*_, then ${\bar{X}}_{A}$ Granger causes ${\bar{X}}_{B}$;
      (b) if ${\bar{X}}_{A}$ does not Granger cause ${\bar{X}}_{B}$, then *X*_*A*_ Granger causes *X*_*B*_.

Remark 4 If *X*_*B*_ is not noisy [*N*_*B*_ = 0], noise associated with the ‘causal variable’ *X*_*A*_ cannot induce spurious Granger causality from *X*_*A*_ to *X*_*B*_, despite possibly complicated dynamics on both *X*_*A*_ and *X*_*B*_. Another special case of interest is provided by the situation where the two processes are actually independent. Then Φ_*AB*_ = 0, and so the relevant transfer function $\Phi_{AB}\Phi_{BB}^{-1}$ with or without noise added remains zero and there is no causality introduced through the addition of noise.

We comment that our conclusions are at variance with those of Solo (2007), who asserts that addition of both noise sequences *N*_*A*_,*N*_*B*_ to *X*_*A*_,*X*_*B*_ where *X*_*A*_ does not Granger cause *X*_*B*_ means that ${\bar{X}}_{A}$ does not Granger cause ${\bar{X}}_{B}$. There appears to be an unjustified assumption in his work (as confirmed in private communication) where he constructs a triangular spectral factor for the $\bar{X}$ process but does not ensure that the off diagonal term is guaranteed to be stable–stability is simply assumed automatically. Such stability would be a necessary condition for asserting that ${\bar{X}}_{A}$ does not Granger cause ${\bar{X}}_{B}$.

## 5 Signal‐To‐Noise Ratio and Granger Causality

We argue a form of continuity result. If there is additive noise perturbing an arrangement where there is absence of causality, then although generically absence of causality will be lost, we shall show that in a certain sense made more precise below, the introduced degree of non‐causality is small. The practical effect of this result is that small amounts of noise in a particular situation may well be tolerable.

Our starting point is the following observation.

Lemma 2 Consider a complex matrix function *M*(*z*), analytic in *ρ* < |*z*| < *ρ* ^−1^, 0 < *ρ* < 1 with *M*(*z*) = *M* ^⊤^(*z* ^−1^), and positive definite on |*z*| = 1. Suppose

(23) $$M(z)=\sum_{i=-\infty }^{\infty }m_{i}z^{i},\;m_{i}=m_{-i}^{⊤}\in R^{d\times d}$$

and define the causal and anticausal parts by

(24) $$M_{+}(z)=\frac{1}{2}m_{0}+\sum_{i=1}^{\infty }m_{i}z^{i}\;\text{and}\;M_{-}(z)=\frac{1}{2}m_{0}+\sum_{i=-\infty }^{-1}m_{i}z^{i}.$$

Then the matrix function *L*(*z*): = *I* + *ϵM*(*z*) is analytic in *ρ* < |*z*| < *ρ* ^−1^, with *L*(*z*) = *L* ^⊤^(*z* ^−1^), and positive definite on |*z*| = 1. Further to first order in *ϵ* > 0, there holds

(25) $$L=I+\epsilon M\approx (I+\epsilon M_{+})(I+\epsilon M_{-})$$

with *I* + *ϵM*_+_ stable and miniphase.

We remark that the terminology ‘to first order in *ϵ*’ is shorthand for saying that the *L*_2_ norm of the error between *L* above and the approximation of it on the right‐hand side of (25), call it Δ(*z*), is of order *ϵ* ^2^ . The square of this *L*_2_ norm can be computed with the aid of an integration of around the unit circle, as $\text{trace}\frac{1}{2\pi }\int {\left[\Delta (\exp(j\omega ))\right]}^{2}d\omega$ or by taking the squared sum of the coefficients in the Laurent series of the error, that is, $\sum_{-\infty }^{\infty }\text{tr}[\delta_{i}\delta_{i}^{⊤}\;]$.

We will use this result to show that small perturbations in a spectrum give small perturbations in the associated spectral factors, and thence conclude that Granger causality is in a sense continuously dependent on the noise spectrum, it being absent when there is no noise. Accordingly we consider the arrangement studied in the previous section, with the introduction of a scaling parameter on the noise: thus $X={\left[X_{A}^{⊤}\;X_{B}^{⊤}\right]}^{⊤}$ and *X*_*A*_ does not Granger cause *X*_*B*_ nor does it cause *X*_*B*_ instantaneously. The canonical factor *W*(*z*) for the noise‐free spectrum Φ_*XX*_(*z*) is upper block triangular and the innovations covariance matrix *Q* is block diagonal, and they obey the fundamental spectral factorization Eq. 4. Assume that *ϵ* ^1/2^ *N*_*B*_ for some *ϵ* > 0 is a noise process additively perturbing *X*_*B*_, thus

$${\bar{X}}_{B}=X_{B}+\epsilon^{1/2}N_{B},\;\;\Phi_{\bar{B}\bar{B}}=\Phi_{BB}+\epsilon \Phi_{N_{B}N_{B}}.$$

(We have effectively previously dealt with the effect of having a noise process *N*_*A*_ perturbing *X*_*A*_–the noisy process *X*_*A*_ + *N*_*A*_ is known to inherit the property of not Granger causing *X*_*B*_, and so no further consideration is given to *N*_*A*_ and for convenience we take it as zero).

Now note that

(26) $$\Phi_{\bar{X}\bar{X}}=\Phi_{XX}+\epsilon \Phi_{NN}.$$

The spectrum $\Phi_{\bar{X}\bar{X}}$ gives rise to a canonical spectral factor, call it $\bar{W}(z)$ and an associated covariance matrix, call it $\bar{Q}$, satisfying

(27) $$\Phi_{\bar{X}\bar{X}}(z)=\bar{W}(z)\bar{Q}{\bar{W}}^{⊤}(z^{-1}).$$

Our first result follows.

Theorem 6 Adopt the same hypothesis as in Theorem 1 and let *N*_*B*_ be a stationary process with rational spectrum, with the same dimension as *X*_*B*_ , and with *X*,*N*_*B*_ independent. For fixed positive *ϵ*, define ${\bar{X}}_{B}=X_{B}+\epsilon^{1/2}N_{B}$ so that $\Phi_{\bar{X}\bar{X}}=\Phi_{XX}+\epsilon \Phi_{NN}$ where the (1,1), (1,2), (2,1) blocks of Φ_*NN*_ are zero, and the (2,2) block is $\Phi_{N_{B}N_{B}}$. Let *W*(*z*),*Q* with *W*(*z*) upper block triangular and *Q* block diagonal and $\bar{W}(z),$ $\bar{Q}$ define canonical spectral factorizations of Φ_*XX*_(*z*) and $\Phi_{\bar{X}\bar{X}}(z)$ as in (4) and (27) respectively. Then

1. $\bar{W}(z)-W(z)$ is *O*(*ϵ*) on |*z*| = 1;
2. $\bar{Q}-Q$ is *O*(*ϵ*);
3. $\Phi_{A\bar{B}}\;\Phi_{\bar{B}\bar{B}}^{-1}-\Phi_{AB}\;\Phi_{BB}^{-1}$ is *O*(*ϵ*) on |*z*| = 1, and the anticausal part of $\Phi_{A\bar{B}}\;\Phi_{\bar{B}\bar{B}}^{-1}$ is *O*(*ϵ*) on |*z*| = 1;
4. for suitably small *ϵ*, ${\bar{W}}_{22}(z)$ is minimum phase.

We remark that the first and third bounds imply bounds on the *L*_2_ norms of the quantities which are also *O*(*ϵ*). Evidently, the $\bar{X}$ process is ‘close to’ a process in which *X*_*A*_ does not cause ${\bar{X}}_{B}$ in two senses: the canonical spectral factor is close to upper block triangular with the innovations covariance matrix being block diagonal, and (separately), the anti‐causal part of the two‐sided Wiener filter associated with predicting *X*_*A*_ from ${\bar{X}}_{B}$ has small magnitude on |*z*| = 1 and in *L*_2_ norm.

In the above theorem, we focused on the changes to transfer functions and to the innovations covariance caused by the introduction of noise. It is also relevant to compare the prediction error variances when *X*_*A*_(*s*),*s* ≤ *t*,*X*_*B*_(*s*),*s* < *t* and *X*_*A*_(*s*),*s* ≤ *t*,$X_{\bar{B}}(s),$ *s* < *t* are used to predict *X*_*B*_ and ${\bar{X}}_{B}$ respectively. The results are summarized Theorem 7. It shows that the prediction error ‘measure’ of Granger causality is *O*(*ϵ* ^2^).

Theorem 7 Adopt the same hypothesis as in Theorem 6 and assume that *ϵ* > 0 is sufficiently small that ${\bar{W}}_{22}$ is minimum phase. Then there exist positive *R*,*R* ^*′*^ of *O*(*ϵ* ^2^) for which there hold the upper and lower bounds:

(28) $$\begin{array}{c} \mathrm{Var}\left[{\bar{X}}_{B}(t)-E[{\bar{X}}_{B}(t)\;|\;{\bar{X}}_{B}(s):s<t]\right]\geq R+{\bar{Q}}_{22}-{\bar{Q}}_{12}^{⊤}{\bar{Q}}_{11}^{-1}{\bar{Q}}_{12}\\ =R+\mathrm{Var}\left[{\bar{X}}_{B}(t)-E[{\bar{X}}_{B}(t)\;|\;X_{A}(t),X_{A}(s),{\bar{X}}_{B}(s):s<t]\right] \end{array}$$

and

(29) $$\mathrm{Var}\left[{\bar{X}}_{B}(t)-E[{\bar{X}}_{B}(t)\;|\;{\bar{X}}_{B}(s):s<t]\right]\leq (1+R^{\prime })\mathrm{Var}\left[{\bar{X}}_{B}(t)-E[{\bar{X}}_{B}(t)\;|\;X_{A}(t),X_{A}(s),{\bar{X}}_{B}(s):s<t]\right]\;.\;$$

## 6 Effect of Filtering on Granger Causality

Consider a stationary full‐rank process $X={\left[X_{A}^{⊤}\;X_{B}^{⊤}\right]}^{⊤}$. Instead of observing processes *X*_*A*_,*X*_*B*_, we observe the process

(30) $$\left[\begin{array}{c} {\bar{X}}_{A}(t)\\ {\bar{X}}_{B}(t) \end{array}\right]=T(L)\left[\begin{array}{c} X_{A}(t)\\ X_{B}(t) \end{array}\right]\;\;T(L):=\left[\begin{array}{cc} T_{A}(L) & T_{AB}(L)\\ T_{BA}(L) & T_{B}(L) \end{array}\right]\;$$

where *T*(*L*) is a causal transfer function, partitioned conformably with ${\left[X_{A}^{⊤}\;X_{B}^{⊤}\right]}^{⊤}$. We consider the question: if *X*_*A*_ does not Granger cause *X*_*B*_, will ${\bar{X}}_{A}$ not Granger cause ${\bar{X}}_{B}$? Conversely, and on occasions more importantly, if one observes that ${\bar{X}}_{A}$ does not Granger cause ${\bar{X}}_{B}$, can one conclude that *X*_*A*_ does not Granger cause *X*_*B*_? Questions of this type go back some time, see for example, Pierce and Haugh (1977), Solo, (2007, 2016) , Florin et al. (2013), Barnett and Seth (2011), and Seth et al. (2013).

In the following theorem, we give a general necessary and sufficient condition for ${\bar{X}}_{A}$ not to cause ${\bar{X}}_{B}$ in the sense of Granger.

Theorem 8 Suppose Assumption 1 holds, and let ${\left[{\bar{X}}_{A}^{⊤}(t)\;{\bar{X}}_{B}^{⊤}(t)\right]}^{⊤}$ be defined by () where *T*(*L*) is a causal stable miniphase transfer function such that *T*(0) = *I*_*d*_. Then, ${\bar{X}}_{A}$ does not Granger cause ${\bar{X}}_{B}\;$if and only if 

(31) $$\Pi_{21}(L)T^{A}(L)+\Pi_{22}(L)T^{BA}(L)=0\;$$

where

(32) $$\Pi (L)=W{\left(L\right)}^{-1}=\left[\begin{array}{cc} \Pi_{11}(L) & \Pi_{12}(L)\\ \Pi_{21}(L) & \Pi_{22}(L) \end{array}\right]\;\text{and}\;T{\left(L\right)}^{-1}=\left[\begin{array}{cc} T^{A}(L) & T^{AB}(L)\\ T^{BA}(L) & T^{B}(L) \end{array}\right]$$

are partitioned conformably with ${\left[X_{A}^{⊤}\;X_{B}^{⊤}\right]}^{⊤}$.

Provided Π_22_(*z*) is miniphase, condition (31) shows it is generally possible to choose the filter *T*(*L*) so that ${\bar{X}}_{A}$ does not Granger cause ${\bar{X}}_{B}$, irrespective of the causal relation between *X*_*A*_ and *X*_*B*_. Filtering can make Granger causality invisible. In general, Granger causality from *X*_*A*_ to *X*_*B*_ is **not invariant** to the application of a multivariate causal miniphase filter to *X*(*t*). When condition (31) holds, ${\bar{X}}_{A}$ does not Granger cause ${\bar{X}}_{B}$ even if *X*_*A*_ does Granger cause *X*_*B*_. Conversely, if Π_12_(*L*)= 0, so *X*_*A*_ does not Granger cause *X*_*B*_, (31) does not hold if we have Π_22_(*L*)*T* ^*BA*^(*L*) ≠ 0 : *X*_*A*_ does not Granger cause *X*_*B*_, and ${\bar{X}}_{A}$ does Granger cause ${\bar{X}}_{B}$. For example, for a bivariate system (*d* = 2), the latter situation happens when

(33) $$\Pi (L)=I_{2}\;\text{and}\;T(L)=\left[\begin{array}{cc} 1 & 0\\ 0.5\;L & 1 \end{array}\right]\;;$$

here *X*(*t*) = *ϵ*(*t*) and

(34) $${\bar{X}}_{A}(t)=X_{A}(t)=\epsilon_{A}(t)\;\;{\bar{X}}_{B}(t)=(0.5)X_{A}(t-1)+X_{B}(t)=(0.5){\bar{X}}_{A}(t-1)+\epsilon_{B}(t).$$

Barnett and Seth (2011 Abstract, Sections 1 and 3) claim that ‘G‐causality for a stationary vector autoregressive (VAR) process is fully invariant under the application of an arbitrary invertible filter’. The above counterexample shows clearly this claim should be qualified. Conditions under which this type of invariance hold are given by Theorem 8.

In the following corollary, we consider the important case where *T*(*L*) has a block triangular form.

Corollary 1 Under the assumptions of Theorem 8, the following equivalences hold. If *T*_*BA*_(*L*) = 0,

(35) $$X_{A}\;\text{does not Granger cause}\;X_{B}\;\text{if and only if}\;{\bar{X}}_{A}\;\text{does not Granger cause}\;{\bar{X}}_{B}.$$

If *T*_*AB*_(*L*) = 0,

(36) $${\bar{X}}_{A}\;\text{does not Granger cause}\;{\bar{X}}_{B}\;\text{if and only if}\;\Pi_{21}(L)=\Pi_{22}(L)T_{B}{\left(L\right)}^{-1}T_{BA}(L).$$

Remark 5 (35) in Theorem 8 means that Granger causality from *X*_*A*_ to *X*_*B*_ is unaffected by linear causal filtering as long as ${\bar{X}}_{B}(t)$ only depends on current and past values of *X*_*B*_(*t*). The filtered series ${\bar{X}}_{A}(t)$ can involve lagged values of both *X*_*A*_(*t*) and *X*_*B*_(*t*). Further, the fact that (35) is an equivalence means that non‐causality between the filtered series does allow one to conclude that the unfiltered series have the same property. Solo (2016) recently considered the special case where *T*_*AB*_(*L*) = 0 and *T*_*BA*_(*L*) = 0, and showed only sufficiency (*X*_*A*_ does not Granger cause *X*_*B*_ implies that ${\bar{X}}_{A}$ does not Granger cause ${\bar{X}}_{B}$); see also Florin et al. (2013), Barnett and Seth (2011) and Seth et al. (2013) for related results.

Remark 6 (36) of Theorem 8 gives a general condition under which Granger causality from *X*_*A*_ to *X*_*B*_ can be suppressed through filtering. In particular, if Π_21_(*z*) is miniphase, we can always choose *T*(*L*) so that ${\bar{X}}_{A}$ does not Granger cause ${\bar{X}}_{B}$. For example, this is achieved by taking *T*_*B*_(*L*) invertible and

(37) $$T_{BA}(L)=T_{B}(L)\Pi_{22}{\left(L\right)}^{-1}\Pi_{21}(L)\;.$$

In particular, by taking *T*_*B*_(*L*) = *I* and *T*_*BA*_(*L*) = Π_22_(*L*)^−1^Π_21_(*L*), we find that the filter

(38) $$T(L)=\left[\begin{array}{cc} I & 0\\ \Pi_{22}{\left(L\right)}^{-1}\Pi_{21}(L) & I \end{array}\right]$$

eliminates Granger causality from *X*_*A*_ to ${\bar{X}}_{B}$: ${\bar{X}}_{B}$ is ‘orthogonal’ to *X*_*A*_ in the Granger sense.

Remark 7 The assumption that *W*(*L*) is miniphase entails that both *W*_*A*_(*L*) and *W*_*B*_(*L*) are miniphase when *W*_*BA*_(*L*) = 0, for then $\det[W(z)]=\det[W_{A}(z)]\det[W_{B}(z)]$. There is a heuristic explanation of why the introduction of a non miniphase but stable *W*_*B*_(*z*) might produce causality where previously it did not hold. Suppose *X*_*A*_ does not cause *X*_*B*_, but *X*_*B*_ does cause *X*_*A*_. For a specific example, suppose that *X*_*A*_(*t*) = *X*_*B*_(*t* − 2) + *ϵ*_*A*_(*t*),*X*_*B*_(*t*) = *ϵ*_*B*_(*t*), with *ϵ*_*A*_,*ϵ*_*B*_ independent white noise sequences. Now suppose that *X*_*B*_ is subject to processing by a filter which delays it. It is well understood in signal processing theory that any non‐minimum phase filter introduces a delay; a particularly evident example is the filter with transfer function *z* ^*p*^, which produces a delay of *p* units. Suppose *p* is say 3. Then $X_{A}(t)={\bar{X}}_{B}(t+1)+\epsilon_{A}(t)$, so future values of ${\bar{X}}_{B}$ are correlated with past values of *X*_*A*_, which means precisely that *X*_*A*_ does cause ${\bar{X}}_{B}$.

Remark 8 Consider the case where *X*_*A*_ defines a filter which is simply a delay. Then the theorem says that if *X*_*A*_ does not Granger cause *X*_*B*_, the same will be true if *X*_*A*_ is replaced by a delayed version of itself. This can of course be argued from first principles also.

## 7 Effect of Subsampling on Granger Causality

A further question raised in the work of Solo (2007) deals with subsampling. Suppose that a process *X*_*A*_ does not Granger cause *X*_*B*_ nor does it cause *X*_*B*_ instantaneously. Suppose the two processes are subsampled. Will the absence of Granger causality continue to hold for the subprocesses?

As we show here, again appealing to Theorem 3, absence of Granger causality may be lost, and Granger causality may arise in the subsampled processes.

By way of brief comment, we note a refinement of the question. Subsampling of two processes at the same rate may not occur synchronously. Thus, for example, samples of *X*_*A*_ with even time index might be considered along with samples of *X*_*B*_ with odd time index. Also, subsampling may occur at different rates. In fact, it could be that *X*_*A*_ is not subsampled, while *X*_*B*_ is. We will not consider these variants in this section.

We first note a very easily established theorem on the transformation of spectra under subsampling (see Hannan, 1970).^4^

Theorem 9 Let Φ_*XX*_(*z*) denote the spectrum of a process *X*, and suppose the process is sampled (synchronously in the case of a multivariate process) every *M* time units for some positive integer *M* , to generate a process $\bar{X}$, defined for time instants …,−*M*,0,*M*,2*M*,…. Then the spectrum of $\bar{X}$, defined using covariance data with lags which are integer multiples of *M* as

(39) $$\Phi_{\bar{X}\bar{X}}(z^{M})=\sum_{k=-\infty }^{\infty }z^{Mk}E[X(0)X^{⊤}(-kM)]$$

is expressible as

(40) $$\Phi_{\bar{X}\bar{X}}(z^{M})=\frac{1}{M}\sum_{i=0}^{M-1}\Phi_{XX}(\omega^{i}z)$$

where $\omega =\exp(\sqrt{-1}2\pi /M)$.

The proof is an immediate consequence of the fact that

(41) $$\Phi_{XX}(\omega^{i}z)=\sum_{k=-\infty }^{\infty }{\left(\omega^{i}z\right)}^{k}E[X(0)X^{⊤}(-k)]$$

and the addition of these equations for *i* = 0,…,*M* − 1.

In our counterexample, we shall take *M* = 2. This means that $\Phi_{\bar{X}\bar{X}}(z)=\Phi_{XX}(z)+\Phi_{XX}(-z)$. In detail, consider a process *X* defined by a unit matrix for the innovations covariance, and a canonical spectral factor given by

(42) $$W(z)=\left[\begin{array}{cc} {}*** & \frac{z}{1-\frac{z}{2}}\\ 0 & \frac{1-\frac{z}{\sqrt{2}}}{1-\frac{z}{2}} \end{array}\right]$$

with the particular expression for the (1,1) entry not being provided, since it turns out to be irrelevant to the calculation. From this expression, it follows that the (1,2) and (2,2) entries of Φ_*XX*_ are:

(43) $$\Phi_{AB}(z)=\left(\frac{z}{1-\frac{z}{2}}\right)\left(\frac{1-\frac{z^{-1}}{\sqrt{2}}}{1-\frac{z^{-1}}{2}}\right)=\frac{z-\frac{1}{\sqrt{2}}}{\frac{5}{4}-\frac{1}{2}(z+z^{-1})}\;\;$$

(44) $$\Phi_{BB}(z)=\left(\frac{1-\frac{z}{\sqrt{2}}}{\left(1-\frac{z}{2}\right)}\right)\left(\frac{1-\frac{z^{-1}}{\sqrt{2}}}{\left(1-\frac{z^{-1}}{2}\right)}\right)=\frac{\frac{3}{2}-\frac{1}{\sqrt{2}}(z+z^{-1})}{\frac{5}{4}-\frac{1}{2}(z+z^{-1})}.$$

Based on the formula for subsampled spectra, we now have:

(45) $$\Phi_{Ā\bar{B}}(z^{2})=\frac{z-\frac{1}{\sqrt{2}}}{\frac{5}{4}-\frac{1}{2}(z+z^{-1})}+\frac{-z-\frac{1}{\sqrt{2}}}{\frac{5}{4}+\frac{1}{2}(z+z^{-1})}=\frac{(1-\frac{5}{2\sqrt{2}})+z^{2}}{\left[\frac{5}{4}-\frac{1}{2}(z+z^{-1})][\frac{5}{4}+\frac{1}{2}(z+z^{-1})\right]}\;$$

(46) $$\Phi_{\bar{B}\bar{B}}(z^{2})=\frac{\frac{3}{2}-\frac{1}{\sqrt{2}}(z+z^{-1})}{\frac{5}{4}-\frac{1}{2}(z+z^{-1})}+\frac{\frac{3}{2}+\frac{1}{\sqrt{2}}(z+z^{-1})}{\frac{5}{4}+\frac{1}{2}(z+z^{-1})}=\frac{\left[\frac{15}{8}-\sqrt{2}-\frac{1}{\sqrt{2}}(z^{2}+z^{-2})\right]}{\left[\frac{5}{4}-\frac{1}{2}(z+z^{-1})][\frac{5}{4}+\frac{1}{2}(z+z^{-1})\right]}\;$$

hence

(47) $$\Phi_{Ā\bar{B}}(z^{2})\Phi_{\bar{B}\bar{B}}^{-1}(z^{2})=\frac{(1-\frac{5}{2\sqrt{2}})+z^{2}}{\frac{15}{8}-\sqrt{2}-\frac{1}{\sqrt{2}}(z^{2}+z^{-2})}=\frac{z^{4}+(1-\frac{5}{2\sqrt{2}})z^{2}}{-\frac{1}{\sqrt{2}}z^{4}+(\frac{15}{8}-\sqrt{2})z^{2}-\frac{1}{\sqrt{2}}}.$$

The denominator polynomial has four zeros on the unit circle (at $\pm 0.81\pm 0.58\sqrt{-1})$ and there are no cancellations with the zeros of the numerator polynomial (at 0,0,$\pm 0.876\sqrt{-1}$ ). (The presence of unit circle zeros rather than zeros inside the unit circle is not of particular note. One can also verify that if the (2,2) term of the spectral factor in the example is changed to be $(1-\frac{z}{3}){\left(1-\frac{z}{2}\right)}^{-1}$, then the denominator polynomial has two zeros inside and two outside the unit circle, at ±1.382 and ±0.869.) So in both cases the transfer function is not causal. *This means that the downsampling destroys the absence of Granger causality*.

This result is not consistent with the claim of Solo (2007). It would appear that in seeking to construct a canonical spectral factorization for the subsampled process, he forces triangularity (which is needed to conclude absence of Granger causality) but in the process cannot assure that the proposed spectral factor remains stable, that is, the simultaneous requirements on the spectral factor for it to be triangular and stable cannot both be met. The fact that an underlying process may have unidirectional Granger causality, while a time aggregated version of it (a form of subsampling) has bidirectional Granger causality, has also been observed in Chambers and McCrorie (2004).

## 8 Conclusion

In this article, we have sought to explain how intuition can be misleading in determining the effect of certain changes made to an underlying model on the Granger causality properties associated with that model. For the important case of additive noise, we have established general conditions under which additive noise does not affect non‐causality properties, as well as conditions under which noise induces spurious Granger causality. It is clear from these that additive noise generally distorts causal relations, even though there are interesting cases where it does not. For example, in the case of measurement errors, if *X*_*B*_ is not noisy, noise on *X*_*A*_ does not induce spurious Granger causality from *X*_*A*_ to *X*_*B*_. To derive our results, we mostly rely on generating function methods, canonical spectral factors, Wold decompositions and spectra. These approaches considerably simplify many proofs. Besides rigorous demonstrations, our findings extend and qualify the early results of Newbold (1978), which focused on special cases. Further, in our discussion of noise, we have drawn attention to the usefulness of thinking of an ‘amount’ of causality: if noise is ‘low’, the distortion to the predictability and Granger causal properties of the process is also ‘low’.

We have also revisited the effects of linear transformations, filtering and subsampling on Granger causality properties. These results include general necessary and sufficient conditions under which ‘spurious’ causal relations between time series are not induced by linear transformations of the variables involved. This characterization also yields linear transformations (or filters) which can eliminate Granger causality from one vector to another one. The various results presented allow one to correct some erroneous statements in the earlier literature [as in Solo (2007) and Barnett and Seth (2011)] on Granger causality in the presence of measurement errors and variable transformations.

The fact that additive noise can distort Granger causality relations means that empirical findings on such properties become more delicate to interpret. Indeed, inferences on ‘noisy variables’ remain perfectly valid as long the causal properties are ascribed to observed variables. If we have no theory (such as an economic or a physical model) that can lead one to distinguish between a ‘true’ latent variable and a ‘measured’ variable, there is no error‐in‐variables problem. Otherwise, one must be aware that the causal structure of the original ‘uncontaminated’ variables can be different. In this context, it is quite interesting to observe that no ‘spurious’ causality can arise when noise only affects the output variable (see Theorem 5). Further, when the signal‐to‐noise ratio is large, distortions on apparent causality will also be small (see Theorem 6).

Similarly, the fact that aggregation and subsampling can distort dynamic relations has been widely discussed; see Tiao and Wei (1976), Wallis (1974), Sims (1974), Wei (1982), Hylleberg (1986), Marcellino (1999), Kaiser and Maravall (2001), Breitung and Swanson (2002), Gong et al. (2015), and the references in the survey of Silvestrini and Veredas (2008). For a specific example showing that causal relations are modified by changing observation frequency, see Dufour et al. (2012). However, Theorem 8 and Corollary 1 give general necessary and sufficient conditions under which ‘spurious’ causal relations between (vector) time series will not be induced by linear transformations of the variables involved. This also yields linear transformations (or filters) which can eliminate Granger causality from one vector to another one.

Unaddressed to this point is a corresponding result on subsampling. It is well known that a continuous‐time band‐limited process and a sampled version of it with sampling frequency in excess of twice the approximate ‘cut‐off’ frequency of the continuous‐time process have more or less the same information. This would suggest that Granger causality properties of continuous‐time band‐limited processes would be ‘almost’ preserved if they were sampled frequently enough, that is, in these circumstances the loss of Granger causality would be small. On a possible avenue in this direction, see Pollock (2012).

Due to dependence on an information set, Granger causality properties are not generally invariant to observation frequency. In contrast with contamination by ‘noise’ – which is typically problematic – non‐invariance to observation frequency may not be a ‘problem’ . Indeed, it can have considerable practical meaning and usefulness: different mechanisms may matter for short and long‐run predictability as well as decisions, in the same way that different ‘laws’ apply to micro‐phenomena and macro‐phenomena in physics. For economic and financial decisions, short‐run and long‐decisions may depend on different factors and require different rules. High‐frequency decisions require prediction for data observed at high frequency, while longer‐run decisions require predictability for data observed at low frequencies. Granger causality analysis at different observation frequencies can provide information on this. It would certainly be of interest to develop a systematic framework for exploring and exploiting such features. The use of mixed frequency data [as proposed in Ghysels et al. (2016)] could also be relevant in this context. However these problems clearly go beyond the scope of the present article.

A different area worthy of examination is that of networked systems. The underlying structure could be viewed using a directed graph, An edge from vertex *i* to vertex *j* would mean that process *i* is caused by process *j* . One could begin by examining whether, in a path graph with vertex set {*v*_1_,*v*_2_,…,*v*_*N*_} and edge set {*e*_12_,*e*_23_,…,*e*_(*N* − 1)*N*_}, Granger causality properties of nonadjacent nodes could be predicted from the corresponding properties of adjacent nodes.

The results given in this article rely on the common assumption of (second‐order) stationarity. Even though there is no reason to think distortions induced by measurement errors would suddenly stop to exist in nonstationary setups, it would be of interest to extend our results to nonstationarity setups, for example, through Hilbert space techniques, especially to evaluate whether non‐stationarity tends to increase or decrease the distortions. Note that nonstationary models are explicitly allowed in Dufour and Renault (1998) and Dufour et al. (2006); for other articles which apply Hilbert space methods to Granger causality, see Hosoya (1977), Florens and Mouchart, 1982 (1982, 1985) , Florens et al. (1993), Florens and Fougère (1996), Triacca, (1998, 2000) , Al‐Sadoon (2014). To study measurement errors, careful consideration of the type of nonstationarities is needed, and different proof methods may be required. Another possibly more general approach to incorporate non‐stationarity would consist in working with the underlying probability measures, along the lines suggested by Mykland (1986). We leave such extensions to future work.

## Acknowledgements

This work is supported by the Australian Research Council's Discovery Project DP‐160104500, CSIRO‐Data61, Austrian FWF grant project number P24198‐N18, the William Dow Chair of Political Economy (McGill University), the Bank of Canada (Research Fellowship), the Toulouse School of Economics (Pierre‐de‐Fermat Chair of excellence), the Universitad Carlos III de Madrid (Banco Santander de Madrid Chair of excellence), the Natural Sciences and Engineering Research Council of Canada, the Social Sciences and Humanities Research Council of Canada, the Fonds de recherche sur la société et la culture (Québec).

## Appendix A

**Proof of Lemma 1** Notice first that

(A1) $$\begin{array}{lll} E[Z(t)\;|\;X(s),Y(s):s<t] & = & E[X(t)+Y(t)\;|\;\;X(s),Y(s):s<t]\\ & = & E[X(t)\;|\;X(s),Y(s):s<t]+E[Y(t)\;|\;X(s),Y(s):s<t]\\ & = & E[X(t)\;|\;X(s):s<t]+E[Y(t)\;|\;Y(s):s<t] \end{array}$$

Accordingly, using the fact that the space spanned by *Z*(*s*) = *X*(*s*) + *Y*(*s*) for *s* < *t* is a subspace of the space spanned by *X*(*s*),*Y*(*s*) for *s* < *t* , we have

(A2) $$\begin{array}{lll} \mathrm{Var}[Z(t)\;|\;Z(s):s<t] & \geq & \mathrm{Var}[X(t)+Y(t)\;|\;X(s),Y(s):s<t]\\ & = & \mathrm{Var}[X(t)\;|\;X(s):s<t]+\mathrm{Var}[Y(t)\;|\;Y(s):s<t] \end{array}$$

as claimed. Of course the independence of the processes *X*,*Y* is used at the second last step.

**Proof of Theorem 3** First, consider the question of absence of Granger causality and instantaneous causality and assume that condition 2 of Theorem 1 holds. Using (4), (10) and the fact that *W*_21_ = 0, it is easily checked that

(A3) $$\Phi_{AB}\Phi_{BB}^{-1}=W_{12}W_{22}^{-1}.$$

Also, the triangular structure of *W* and its minimum phase character imply that $W_{22}^{-1}$ is stable. Hence $\Phi_{AB}\Phi_{BB}^{-1}$ is also stable. It also assumes the value 0 at *z* = 0 because of the normalization condition *W*(0) = *I*.

To prove the converse, consider a canonical spectral factorization of the type of (4) (with no *apriori* restriction on the block triangular structure of *W*). Suppose that $\Phi_{AB}\Phi_{BB}^{-1}:=T(z)$ is stable and zero at *z* = 0. We first assert that this will imply the condition *W*_21_ = 0 in the minimum phase, stable spectral factor normalized to be *I* at *z* = 0. To see this, suppose first that *V*_*B*_(*z*) is a minimum phase, stable transfer function with *V*_*B*_(*∞*) = *I* and *R*_*B*_ is a positive definite matrix such that

(A4) $$\Phi_{BB}(z)=V_{B}(z)R_{B}V_{B}^{⊤}(z^{-1}).$$

Define

(A5) $$V_{AB}(z)=\Phi_{AB}(z)\Phi_{BB}^{-1}(z)V_{B}(z).$$

Note that *V*_*AB*_(*z*) is then stable and zero at *z* = 0. Note also that the last two equations yield

(A6) $$\Phi_{AB}(z)=V_{AB}(z)R_{B}V_{B}^{⊤}(z^{-1}).$$

By the fact that Φ_*XX*_ is positive definite for all *z* = *e* ^*jω*^, it follows that $\Phi_{AA}-\Phi_{AB}\Phi_{BB}^{-1}\Phi_{BA}$ is also positive definite for all *z* = *e* ^*jω*^, and accordingly, we can define a minimum phase, stable transfer function *V*_*A*_(*z*) with *V*_*A*_(0) = *I* and a positive definite *R*_*A*_ such that

(A7) $$\Phi_{AA}(z)-\Phi_{AB}(z)\Phi_{BB}^{-1}(z)\Phi_{BA}(z)=V_{A}(z)R_{A}V_{A}^{⊤}(z^{-1}).$$

Observe that

(A8) $$\Phi_{AB}(z)\Phi_{BB}^{-1}(z)\Phi_{BA}(z)=\Phi_{AB}(z)\Phi_{BB}^{-1}(z)[V_{B}(z)R_{B}V_{B}^{⊤}(z^{-1})]\Phi_{BB}^{-1}(z)\Phi_{BA}(z)=V_{AB}(z)R_{B}V_{AB}^{⊤}(z^{-1})$$

or equivalently

(A9) $$\Phi_{AA}(z)=V_{AB}(z)R_{B}V_{AB}^{⊤}(z^{-1})+V_{A}(z)R_{A}V_{A}^{⊤}(z^{-1}).$$

Now from equations (A9), (A6) and (A4), we have

(A10) $$\left[\begin{array}{cc} \Phi_{AA}(z) & \Phi_{AB}(z)\\ \Phi_{BA}(z) & \Phi_{BB}(z) \end{array}\right]=\left[\begin{array}{cc} V_{A}(z) & V_{AB}(z)\\ 0 & V_{B}(z) \end{array}\right]\left[\begin{array}{cc} R_{A} & 0\\ 0 & R_{B} \end{array}\right]\left[\begin{array}{cc} V_{A}^{⊤}(z^{-1}) & 0\\ V_{AB}^{⊤}(z^{-1}) & V_{B}^{⊤}(z^{-1}) \end{array}\right].$$

Now make the definitions

(A11) $$V(z)=\left[\begin{array}{cc} V_{AA}(z) & V_{AB}(z)\\ 0 & V_{BB}(z) \end{array}\right]\;\;\;R=\left[\begin{array}{cc} R_{A} & 0\\ 0 & R_{B} \end{array}\right]\;\;$$

and observe that *V*(*z*) is stable, minimum phase and has *V*(0) = *I*. Accordingly, *V*(*z*) is the unique miniphase stable spectral factor with *V*(0) = *I*, and condition 2 of Theorem 1 is evidently fulfilled.

The argument for Granger causality only is similar but not identical, and so we present the details. Assume then there is an absence of Granger causality, which means that there is a spectral factorization with canonical spectral factor *W*(*z*) and not necessarily block diagonal innovations covariance matrix of the form

(A12) $$\Phi_{XX}(z)=\left[\begin{array}{cc} W_{11}(z) & W_{12}(z)\\ 0 & W_{22}(z) \end{array}\right]\left[\begin{array}{cc} Q_{11} & Q_{12}\\ Q_{12}^{⊤} & Q_{22} \end{array}\right]\left[\begin{array}{cc} W_{11}^{⊤}(z^{-1}) & 0\\ W_{12}^{⊤}(z^{-1}) & W_{22}^{⊤}(z^{-1}) \end{array}\right].$$

It then follows that

(A13) $$\Phi_{AB}\Phi_{BB}^{-1}=(W_{11}Q_{12}+W_{12}Q_{22})W_{22}^{-1}$$

and this is evidently stable.

For the converse, we follow the proof applying for the case where the aim was to conclude an absence of Granger causality and instantaneous causality. The proof applies with the first change that *V*_*AB*_(*z*) is no longer guaranteed to be zero at *z* = 0. Equation (A10) holds, including the fact that *R* is block diagonal, but *V*(*∞*) ≠ *I*, due to the generally nonzero nature of *V*_*AB*_(0). Now by setting

(A14) $$W(z)=\left[\begin{array}{cc} I & -V_{AB}(0)\\ 0 & I \end{array}\right]V(z)$$

equation (A12) arises, and Granger causality is then proved.

**Proof of Theorem 4** For the first claim, let *W*_11_(*z*),*W*_12_(*z*),*W*_22_(*z*),*Q*_11_, *Q*_12_ and *Q*_22_ be the matrices of the canonical spectral factor description of the joint spectrum of *X*_*A*_,*X*_*B*_. If *W*_12_ = 0, then it is evident that the two processes are independent. So we must prove that if *W*_12_ ≠ 0, then *X*_*B*_ Granger causes *X*_*A*_. This is equivalent to showing that

(A15) $$\mathrm{Var}[X_{A}(t)\;|\;X_{A}(s),X_{B}(s):s<t]\leq \mathrm{Var}[X_{A}(t)\;|\;X_{A}(s):s<t]$$

and the two conditional covariance matrices are unequal. Now observe that from the canonical factorization of the joint process *X*_*A*_,*X*_*B*_, we have immediately

(A16) $$\mathrm{Var}[X_{A}(t)\;|\;X_{B}(t),X_{A}(s),X_{B}(s):s<t]=Q_{11}.$$

Further, we know that *X*_*A*_(*t*) = *W*_11_(*L*)*ϵ*_*A*_(*t*) + *W*_12_(*L*)*ϵ*_*B*_(*t*). This expresses *X*_*A*_ as a sum of two independent processes and the lemma above applies. Note that the variance of the one step ahead prediction for the process *W*_11_(*L*)*ϵ*_*A*_(*t*) is precisely *Q*_1_, since *W*_11_(*z*) is a canonical spectral factor. Hence the claim (A15) is established.For the second part, we are required to show that when *X*_*A*_ does not cause *X*_*B*_, either the processes are independent or

(A17) $$\mathrm{Var}[X_{A}(t)\;|\;X_{B}(t),X_{A}(s),X_{B}(s):s<t]\leq \mathrm{Var}[X_{A}(t)\;|\;X_{A}(s):s<t]$$

where equality does not hold. Let *Q*_12_ be the (1, 2) block of the matrix *Q* in the canonical spectral factorization; in general, it is not zero. Then it is easily seen that

(A18) $$\mathrm{Var}[X_{A}(t)\;|\;X_{B}(t),X_{A}(s),X_{B}(s):s<t]=Q_{11}-Q_{12}Q_{22}^{-1}Q_{12}^{⊤}.$$

Next observe that

(A19) $$X_{A}(t)=W_{11}(L)[\epsilon_{A}(t)-Q_{12}Q_{22}^{-1}\epsilon_{B}(t)]+[W_{11}(L)Q_{12}Q_{22}^{-1}+W_{12}(L)]\epsilon_{B}(t).$$

This expresses *X*_*A*_ as the sum of two independent processes, since $\epsilon_{A}(t)-Q_{12}Q_{22}^{-1}\epsilon_{B}(t)$ and *ϵ*_*B*_(*t*) are independent. In the additive decomposition, the second process will evanesce if and only if *W*_12_(*z*) = 0,*Q*_12_ = 0, that is, the two processes *X*_*A*_,*X*_*B*_ are independent. The first process is in fact the process $X_{A}(t)-E[X_{A}(t)$ |*X*_*B*_(*t*),*X*_*A*_(*s*),*X*_*B*_(*s*):*s* < *t*], as is easily checked. Hence once again the lemma applies to yield the result (A17) as required.

**Proof of Theorem 6** The fact that $\Phi_{\bar{X}\bar{X}}$ satisfies (26) implies that

(A20) $$\Phi_{\bar{X}\bar{X}}=WQ^{1/2}[I+\epsilon Q^{-1/2}W^{-1}\Phi_{NN}W^{-*}Q^{-1/2}]Q^{1/2}W^{*}.$$

Now identify *M* in Lemma 2 with *Q* ^−1/2^ *W* ^−1^Φ_*NN*_ *W* ^−∗^ *Q* ^−1/2^ to conclude that a stable and miniphase spectral factor of the noise perturbed spectrum $\Phi_{\bar{X}\bar{X}}$ is (to first order in *ϵ*, corresponding to a low noise situation),

(A21) $$\tilde{W}=WQ^{1/2}[I+\epsilon {\left(Q^{-1/2}W^{-1}\Phi_{NN}W^{-*}Q^{-1/2}\right)}_{+}]$$

in the sense that

(A22) $$\Phi_{\bar{X}\bar{X}}(z)=\tilde{W}(z){\tilde{W}}^{⊤}(z^{-1}).$$

Note that $\tilde{W}(z)$ is not canonical because the requirement $\tilde{W}(0)=I$ is not fulfilled. Define *J* to be the value of

(A23) $$\left[I+\epsilon {\left(Q^{-1/2}W^{-1}\Phi_{NN}W^{-*}Q^{-1/2}\right)}_{+}\right]$$

at *z* = 0. Note that this is precisely *I* + *ϵK*, where 2*K* is necessarily nonnegative being (1/2*π*) times the integral around the unit circle of *Q* ^−1/2^ *W* ^−1^Φ_*NN*_ *W* ^−∗^ *Q* ^−1/2^ and note that *J* − *I* (which is *ϵK*) and thus *J* ^−1^ − *I* are *O*(*ϵ*). Then the canonical spectral factor will be

(A24) $$\bar{W}(z)=\tilde{W}(z)J^{-1}Q^{-1/2}$$

since this assures that (27) will hold where we can identify

(A25) $$\bar{Q}=\left[\begin{array}{cc} {\bar{Q}}_{11} & {\bar{Q}}_{12}\\ {\bar{Q}}_{12}^{⊤} & {\bar{Q}}_{22} \end{array}\right]=Q^{1/2}J^{2}Q^{1/2}.$$

From the calculations immediately preceding the last two equations, it is easy to see that the difference $\bar{W}(z)-W(z)$ will be *O*(*ϵ*), as will ${\bar{Q}}_{12}$ and $Q-\bar{Q}$.

Next we consider the claim concerning the two‐sided Wiener filter for estimating *X*_*A*_ from ${\bar{X}}_{B}$. It is easily seen using (A20) that $\Phi_{BA}(z)\Phi_{BB}^{-1}(z)$ differs for each fixed z=exp(jω) from $\Phi_{\bar{B}A}(z)\Phi_{\bar{B}\bar{B}}^{-1}(z)$ by an amount bounded by *O*(*ϵ*) as *ϵ* goes to zero, and hence the anticausal part will have the same property. Since the anticausal part of $\Phi_{BA}(z)\Phi_{BB}^{-1}(z)$ is actually zero, this means the anticausal part of $\Phi_{BA}(z)\Phi_{BB}^{-1}(z)$ will have an *L*_2_ norm that is *O*(*ϵ*).

To prove the final claim, observe that because *W*(*z*) is block upper triangular and canonical, the submatrix *W*_22_(*z*) is minimum phase. Now the right side of $W_{22}^{-1}(z){\bar{W}}_{22}(z)=I+W_{22}^{-1}(z)[{\bar{W}}_{22}(z)-W_{22}(z)]$ represents a perturbation of I by a stable matrix whose norm is bounded by *O*(*ϵ*) on the unit circle, and accordingly it is minimum phase. Hence the product with *W*_22_(*z*) is also minimum phase, that is, ${\bar{W}}_{22}(z)$ is minimum phase. This completes the proof of the theorem.

**Proof of Theorem 7** We start by expressing ${\bar{X}}_{B}$ as a sum of two independent processes. Thus observe that we can express ${\bar{X}}_{B}$ as

(A26) $${\bar{X}}_{B}(t)=[{\bar{W}}_{22}(L){\bar{Q}}_{12}^{⊤}{\bar{Q}}_{11}^{-1}+{\bar{W}}_{21}(L)]{\bar{\epsilon }}_{A}(t)+{\bar{W}}_{22}(L)[{\bar{\epsilon }}_{B}(t)-{\bar{Q}}_{12}^{⊤}{\bar{Q}}_{11}^{-1}{\bar{\epsilon }}_{A}(t)]$$

in obvious notation. Note that the two processes ${\bar{\epsilon }}_{A}(t)$ and ${\tilde{\epsilon }}_{B}(t)={\bar{\epsilon }}_{B}(t)-{\bar{Q}}_{12}^{⊤}{\bar{Q}}_{11}^{-1}{\bar{\epsilon }}_{A}(t)$ are orthogonal, with covariance matrix

(A27) $$E\{\left[\begin{array}{c} {\bar{\epsilon }}_{A}(t)\\ {\tilde{\epsilon }}_{B}(t) \end{array}\right]\left[{\bar{\epsilon }}_{A}^{⊤}(t)\;{\tilde{\epsilon }}_{B}^{⊤}(t)\right]\}=\left[\begin{array}{cc} {\bar{Q}}_{11} & 0\\ 0 & {\bar{Q}}_{22}-{\bar{Q}}_{12}^{⊤}{\bar{Q}}_{11}^{-1}{\bar{Q}}_{12} \end{array}\right].$$

Let *R* denotes the error variance in estimating the value at time *t* of $\left[{\bar{W}}_{22}(L){\bar{Q}}_{12}^{⊤}{\bar{Q}}_{11}^{-1}+{\bar{W}}_{21}(L)]{\bar{\epsilon }}_{A}(t\right)$ from values for *s* < *t*. Recall also that ${\bar{W}}_{22}(z)$ is minimum phase, and must satisfy ${\bar{W}}_{22}(0)=I$. Accordingly, it is a canonical spectral factor of the spectrum of ${\bar{W}}_{22}(L)[{\bar{\epsilon }}_{B}(t)-{\bar{W}}_{12}^{⊤}{\bar{Q}}_{11}^{-1}{\bar{\epsilon }}_{A}(t)]$. Hence the prediction error covariance associated with estimating the value at time *t* of ${\bar{W}}_{22}(L){\tilde{\epsilon }}_{B}(t)$ from values for *s* < *t* is precisely ${\bar{Q}}_{22}-{\bar{Q}}_{12}^{⊤}{\bar{Q}}_{11}^{-1}{\bar{Q}}_{12}$. We can now use Lemma 1 as we did in the proof of Theorem 4 to conclude that

(A28) $$\mathrm{Var}[{\bar{X}}_{B}(t)-E[{\bar{X}}_{B}(t)\;|\;{\bar{X}}_{B}(s):s<t]]\geq R+{\bar{Q}}_{22}-{\bar{Q}}_{12}^{⊤}{\bar{Q}}_{11}^{-1}{\bar{Q}}_{12}.$$

From the canonical factorization for $\Phi_{\bar{X}\bar{X}}$ we have that the second term on the right in the above equation is given by

(A29) $$\mathrm{Var}[{\bar{X}}_{B}(t)-E[{\bar{X}}_{B}(t)\;|\;X_{A}(t),X_{A}(s),{\bar{X}}_{B}(s):s<t]={\bar{Q}}_{22}-{\bar{Q}}_{12}^{⊤}{\bar{Q}}_{11}^{-1}{\bar{Q}}_{12}.$$

We now turn to establishing the bound on *R*. ${\bar{X}}_{B}(t)$ from ${\bar{X}}_{A}(s),$ *s* ≤ *t*,${\bar{X}}_{B}(s),$ *s* < *t*. This is, as is well known, precisely ${\bar{Q}}_{22}-{\bar{Q}}_{12}^{⊤}{\bar{Q}}_{11}^{-1}{\bar{Q}}_{12}$. That for predicting ${\bar{X}}_{B}(t)$ from ${\bar{X}}_{B}(s)$ is more complicated. To make progress observe that Consider the transfer function acting on ${\bar{\epsilon }}_{A}$ in (A26). From Theorem 6, ${\bar{Q}}_{12}$ and ${\bar{W}}_{21}(z)$ are *O*(*ϵ*) and so the transfer function multiplying ${\bar{\epsilon }}_{A}$ is of order *ϵ*. Hence we see that *R*, being the prediction error variance in estimating a variable whose spectrum is proportional to *ϵ* ^2^ must itself be proportional to *ϵ* ^2^. Hence the increase in prediction error covariance when ${\bar{X}}_{A}$ ceases to be available for estimating ${\bar{X}}_{B}$ is *bounded from below* by a quantity proportional to *ϵ* ^2^.We now derive the overbound of the same order. Choose a constant *R* ^*′*^ so that for all z=exp(jω), there holds

(A30) $$R^{\prime }{\bar{W}}_{22}(z)[{\bar{Q}}_{22}-{\bar{Q}}_{12}^{⊤}{\bar{Q}}_{11}^{-1}{\bar{Q}}_{12}]{\bar{W}}_{22}^{⊤}(z^{-1})\geq [{\bar{W}}_{22}(z){\bar{Q}}_{12}^{⊤}{\bar{Q}}_{11}^{-1}+{\bar{W}}_{21}(z)]{\bar{Q}}_{11}[{\bar{Q}}_{11}^{-1}{\bar{Q}}_{12}{\bar{W}}_{22}^{⊤}(z^{-1})+{\bar{W}}_{21}^{⊤}(z^{-1})].$$

This is possible since for all z=exp(jω) the left side is positive definite. Since the right side is *O*(*ϵ* ^2^), it is clear that *R* ^*′*^ can be taken also as *O*(*ϵ* ^2^). Now ${\bar{X}}_{B}$ is written in (A26) as the sum of two orthogonal processes. Hence the spectrum of ${\bar{X}}_{B}$ will be the sum of the spectra of these two processes, that is,

$${\bar{W}}_{22}(z)[{\bar{Q}}_{22}-{\bar{Q}}_{12}^{⊤}{\bar{Q}}_{11}^{-1}{\bar{Q}}_{12}]{\bar{W}}_{22}^{⊤}(z^{-1})+[{\bar{W}}_{22}(z){\bar{Q}}_{12}^{⊤}{\bar{Q}}_{11}^{-1}+{\bar{W}}_{21}(z)]{\bar{Q}}_{11}[{\bar{Q}}_{11}^{-1}{\bar{Q}}_{12}{\bar{W}}_{22}^{⊤}(z^{-1})+{\bar{W}}_{21}^{⊤}(z^{-1})]$$

for z=exp(jω) and this is overbounded by a spectrum, call it Φ_*CC*_(*z*), with

(A31) $$\Phi_{CC}(z)=(R^{\prime }+1){\bar{W}}_{22}(z)[{\bar{Q}}_{22}-{\bar{Q}}_{12}^{⊤}{\bar{Q}}_{11}^{-1}{\bar{Q}}_{12}]{\bar{W}}_{22}^{⊤}(z^{-1})$$

again for z=exp(jω). Hence there exists a process, call it *X*_*D*_ , which is independent of ${\bar{X}}_{B}$, for which $X_{C}={\bar{X}}_{B}+X_{D}$ and whose spectrum is $\Phi_{CC}(z)-\Phi_{\bar{X}\bar{X}}(z)$. By Lemma 1, the variance of the one step prediction estimate using its own past of the process *X*_*C*_ with spectrum of Φ_*CC*_ overbounds the sum of the variances of the one step prediction estimates of each of ${\bar{X}}_{B}$ and *X*_*D*_. * A fortiori* it overbounds the variance of the one step prediction estimate of ${\bar{X}}_{B}$: thus

(A32) $$(R^{\prime }+1)[{\bar{Q}}_{22}-{\bar{Q}}_{12}^{⊤}{\bar{Q}}_{11}^{-1}{\bar{Q}}_{12}]\geq \mathrm{Var}\left[{\bar{X}}_{B}(t)-E[{\bar{X}}_{B}(t)\;|\;{\bar{X}}_{B}(s):s<t]\right].$$

Equivalently, we can write

(A33) $$\mathrm{Var}\left[{\bar{X}}_{B}(t)-E[{\bar{X}}_{B}(t)\;|\;{\bar{X}}_{B}(s):s<t]\right]\leq (R^{\prime }+1)\mathrm{Var}\left[{\bar{X}}_{B}(t)-E[{\bar{X}}_{B}(t)\;|\;X_{A}(t),X_{A}(s),{\bar{X}}_{B}(s):s<t]\right].$$

This completes the proof of the theorem.

**Proof of Theorem 8** Since the process has an autoregressive representation, we can write:

(A34) $$\Pi (L)X(t)=\Pi (L)T{\left(L\right)}^{-1}T(L)X(t)=\bar{\Pi }(L)\bar{X}(t)=\epsilon (t)$$

where

(A35) $$\bar{\Pi }(L)=\left[\begin{array}{cc} {\bar{\Pi }}_{11}(L) & {\bar{\Pi }}_{12}(L)\\ {\bar{\Pi }}_{21}(L) & {\bar{\Pi }}_{22}(L) \end{array}\right]=\left[\begin{array}{cc} \Pi_{11}(L) & \Pi_{12}(L)\\ \Pi_{21}(L) & \Pi_{22}(L) \end{array}\right]\left[\begin{array}{cc} T^{A}(L) & T^{AB}(L)\\ T^{BA}(L) & T^{B}(L) \end{array}\right],\epsilon (t)=\left[\begin{array}{c} \epsilon_{A}(t)\\ \epsilon_{B}(t) \end{array}\right].$$

In particular,

(A36) $${\bar{\Pi }}_{21}(L)=\Pi_{21}(L)T^{A}(L)+\Pi_{22}(L)T^{BA}(L).$$

Since *ϵ*(*t*) is uncorrelated with the past *X*(*t*), hence also of the past of $\bar{X}(t)$, it follows from Proposition 1 in Boudjellaba et al. (1992) that: ${\bar{X}}_{A}$ does not Granger cause ${\bar{X}}_{B}\;$if and only if ${\bar{\Pi }}_{21}(L)=0$.

**Proof of Corollary 1** If *T*_*BA*_(*L*) = 0, it follows from standard results on the inversion of partitioned matrices that

(A37) $$T{\left(L\right)}^{-1}={\left[\begin{array}{cc} T_{A}(L) & T_{AB}(L)\\ 0 & T_{B}(L) \end{array}\right]}^{-1}=\left[\begin{array}{cc} T_{A}{\left(L\right)}^{-1} & -T_{A}{\left(L\right)}^{-1}T_{AB}(L)T_{B}{\left(L\right)}^{-1}\\ 0 & T_{B}{\left(L\right)}^{-1} \end{array}\right]=\left[\begin{array}{cc} T^{A}(L) & T^{AB}(L)\\ T^{BA}(L) & T^{B}(L) \end{array}\right]\;;$$

see (Harville, 2008, Chapter 8, Theorem 8.5.4). Then, the condition takes the form Π_21_(*L*)*T*_*A*_(*L*)^−1^ = 0, which in view of the invertibility of *T*_*A*_(*L*) is equivalent to Π_21_(*L*) = 0. By Proposition 1 in Boudjellaba et al. (1992), *X*_*A*_ does not Granger cause *X*_*B*_ if and only if Π_21_(*L*) = 0, so that

(A38) $${\bar{X}}_{A}\;\text{does not Granger cause}\;{\bar{X}}_{B}\;\text{if and only if}\;X_{A}\;\text{does not Granger cause}\;X_{B}.$$

If *T*_*AB*_(*L*) = 0, we have

(A39) $$T{\left(L\right)}^{-1}={\left[\begin{array}{cc} T_{A}(L) & 0\\ T_{BA}(L) & T_{B}(L) \end{array}\right]}^{-1}=\left[\begin{array}{cc} T_{A}{\left(L\right)}^{-1} & 0\\ -T_{B}{\left(L\right)}^{-1}T_{BA}(L)T_{A}{\left(L\right)}^{-1} & T_{B}{\left(L\right)}^{-1} \end{array}\right]=\left[\begin{array}{cc} T^{A}(L) & T^{AB}(L)\\ T^{BA}(L) & T^{B}(L) \end{array}\right]\;$$

so that *T* ^*A*^(*L*) = *T*_*A*_(*L*)^−1^ and *T* ^*BA*^(*L*) = −*T*_*B*_(*L*)^−1^ *T*_*BA*_(*L*)*T*_*A*_(*L*)^−1^, and condition (31) takes the form

(A40) $$\Pi_{21}(L)T^{A}(L)+\Pi_{22}(L)T^{BA}(L)=\Pi_{21}(L)T_{A}{\left(L\right)}^{-1}-\Pi_{22}(L)T_{B}{\left(L\right)}^{-1}T_{BA}(L)T_{A}{\left(L\right)}^{-1}=0$$

or, equivalently,

(A41) $$\Pi_{21}(L)=\Pi_{22}(L)T_{B}{\left(L\right)}^{-1}T_{BA}(L).$$

Thus, ${\bar{X}}_{A}$ does not Granger cause ${\bar{X}}_{B}$ if and only if Π_21_(*L*) = Π_22_(*L*)*T*_*B*_(*L*)^−1^ *T*_*BA*_(*L*).

## Footnotes

1 Without the Gaussian assumption, the results presented in this article continue to hold provided conditional expectations are replaced by projections onto the Hilbert space spanned by components of the respective stationary processes.

2 There may be a proof in the literature for *d* ≥ 2, but we are not aware of it. For completeness, a proof appears in the appendix.

3 Solo in a private communication has indicated that an erroneous step in his proof leads to the discrepancy between his and our results.

4 The relation between Granger causality and subsampling could also be studied by postulating a deeper structural model, such as a continuous‐time model; for some earlier work on continuous‐time Granger causality, see Florens and Fougère (1996) and Comte and Renault (1996). We focus here on the standard discrete‐time second‐order stationary model described in Sections 1 and 2.
